# The MAPKKK and MAPKK gene families in banana: identification, phylogeny and expression during development, ripening and abiotic stress

**DOI:** 10.1038/s41598-017-01357-4

**Published:** 2017-04-25

**Authors:** Lianzhe Wang, Wei Hu, Weiwei Tie, Zehong Ding, Xupo Ding, Yang Liu, Yan Yan, Chunlai Wu, Ming Peng, Biyu Xu, Zhiqiang Jin

**Affiliations:** 1 Key Laboratory of Biology and Genetic Resources of Tropical Crops, Institute of Tropical Bioscience and Biotechnology, Chinese Academy of Tropical Agricultural Sciences, Haikou, Hainan 571101 China; 2 School of Life Science and Engineering, Henan University of Urban Construction, Pingdingshan, Henan 467044 China; 3 Key Laboratory of Genetic Improvement of Bananas, Hainan province, Haikou Experimental Station, China Academy of Tropical Agricultural Sciences, Haikou, Hainan 570102 China

## Abstract

The mitogen-activated protein kinase (MAPK) cascade, which is a major signal transduction pathway widely distributed in eukaryotes, has an important function in plant development and stress responses. However, less information is known regarding the MAPKKK and MAPKK gene families in the important fruit crop banana. In this study, 10 MAPKK and 77 MAPKKK genes were identified in the banana genome, and were classified into 4 and 3 subfamilies respectively based on phylogenetic analysis. Majority of MAPKKK and MAPKK genes in the same subfamily shared similar gene structures and conserved motifs. The comprehensive transcriptome analysis indicated that MAPKKK-MAPKK genes is involved in tissue development, fruit development and ripening, and response to abiotic stress of drought, cold and salt in two banana genotypes. Interaction networks and co-expression assays demonstrated that MAPK signaling cascade mediated network participates in multiple stress signaling, which was strongly activated in Fen Jiao (FJ). The findings of this study advance understanding of the intricately transcriptional control of MAPKKK-MAPKK genes and provide robust candidate genes for further genetic improvement of banana.

## Introduction

In response to development and stress stimuli, there are many highly elaborate signaling network involved in sensing and transmitting signal in plants. Among the signaling network, mitogen-activated protein kinases (MAPKs) signaling cascade, which works as a universal signal transduction module transducing various extra- and intracellular signals, is involved in multiple biological processes^1, 2^. The classical MAPK signaling cascade which conserved throughout eukaryotes, is composed of a linear cascade of three specific class of serine/threonine protein kinases: MAPK kinase kinase (MAPKKK), MAPK kinase (MAPKK) and MAPK, and they are linked to upstream and downstream regulators by phosphorylation^3^.

In plants, MAPK signaling pathways participate in the regulation of many biologic processes, such as plant development, growth, ripening, hormonal signaling and in response to a diversity of biotic and abiotic stress^3–5^. To date, several plant MAPK signaling cascades have been characterized in many species. Arabidopsis MEKK1-MKK4/5-MPK3/6 cascade is firstly identified as MAPK signaling module in plants, and they are involved in plant innate immunity of flg22 signal transmission^5, 6^. Then the Arabidopsis MEKK1-MKK1/2-MPK4 and tobacco NPK1-MEK1-Ntf6 were identified to play important roles in biotic stress defense^7, 8^. The Arabidopsis ANP3-MKK6-MPK4, YDA-MKK4/5-MPK3/6 and tobacco NPK1-NQK1/NtMEK1-NRK1 cascades participate in regulating development processes^9, 10^. The MEKK1-MKK2-MPK4 cascade contributes to the abiotic stress tolerance of freezing^11^. In addition, some genes related to MAPK cascade pathway were identified to play a vital role in environmental stress responses. For instance, overexpression of *OsMAPK5* could enhance drought, salt, and cold tolerances in rice^12^. Overexpression of tobacco *NPK1*, belonging to the MAPKKK family, improved maize resistance to drought^13^. Transgenic plants overexpressing *MKK2* activated *RD29A* and *RD29B* resulted in increased salt tolerance in Arabidopsis^14^. Genetic modification of the MAP kinase genes, including *ZmSIMK1*, *CsNMAPK* and *GhMPK2*, enhanced tolerance to high-salinity stress^15–17^. Together, the above evidences support that MAPK cascades pathway is a crucial regulator of plant development and stress response.

The MAPK signaling is conserved in high plants, and many genes involved in MAPK cascade have been identified in several species based on genome sequencing. There are 20 MAPKs, 10 MAPKKs and 80 MAPKKKs in Arabidopsis^3, 18^; 17 MAPKs, 8 MAPKKs and 75 MAPKKKs in rice^19, 20^; 19 MAPKs, 9 MAPKKs and 71 MAPKKK in maize^21, 22^; 16 MAPK, 6 MAPKK and 89 MAPKKK genes in tomato^23, 24^; and 14 MAPKs, 6 MAPKKs and 59 MAPKKKs in cucumber^25^. In banana, 25 MAPKs has been identified and was found to be involved in different stages of fruit ripening previously^26^. However, less evidence is known about the MAPKKK and MAPKK gene family in banana which is the most popular fruit and is vital for food security for millions of people in the world^27^. Due to the limitation of banana cultivation zones, banana is only a staple food for the largely impoverished continent of Africa, so studies of the banana proceeded slowly^27^. In spite of the economic and social importance of banana and the critical role of MAPK signaling cascade in plants, no systematic MAPKKKs and MAPKKs have been characterized in banana. Thus, investigation of these gene families is necessary to elucidate the biological processes of banana development and stress responses.

In this study, we identified 10 *MAPKK* and 77 *MAPKKK* genes from the banana genome, and further investigated their phylogenetic relationship, protein motifs, gene structure, expression patterns in diverse tissues, fruit development and ripening process, and the responses to abiotic stress in two banana varieties. Further, we characterized the interaction networks of MAPKKK-MAPKK-MAPK in response to abiotic stress in banana. This comprehensive study would increase our understanding of MAPKKK and MAPKK associated with developmental process and abiotic stress responses, and build a solid foundation for future studies aimed at MAPK signaling cascade-mediated genetic improvement of banana.

## Results

### Identification and phylogenetic analysis of banana MAPKK and MAPKKK families

To identify all MAPKK and MAPKKK families in the banana, the Hidden Markov Model searches and BLAST searches were performed in the banana genome database using Arabidopsis and rice related sequences as queries. After validating the known conserved domains of the MAPKK and MAPKKK family proteins using the CDD and PFAM databases, a total of 10 MAPKK (MaMKK) and 77 MAPKKK proteins were identified, respectively. The *MaMKK* and *MaMAPKKK* genes were renamed sequentially based on their distribution on chromosomes^24^. The MAPKK and MAPKKK proteins were predicted to encoding 203 (MaMAPKKK8) to 1397 (MaMAPKKK73) amino acid residues, with the relative molecular mass ranging from 23.33 to 155.24 kDa and the theoretical isoelectric points (PIs) ranging from 4.86 (MaMAPKKK38) to 9.84 (MaMKK7) (Supplementary Table S1).

**Figure 1:**
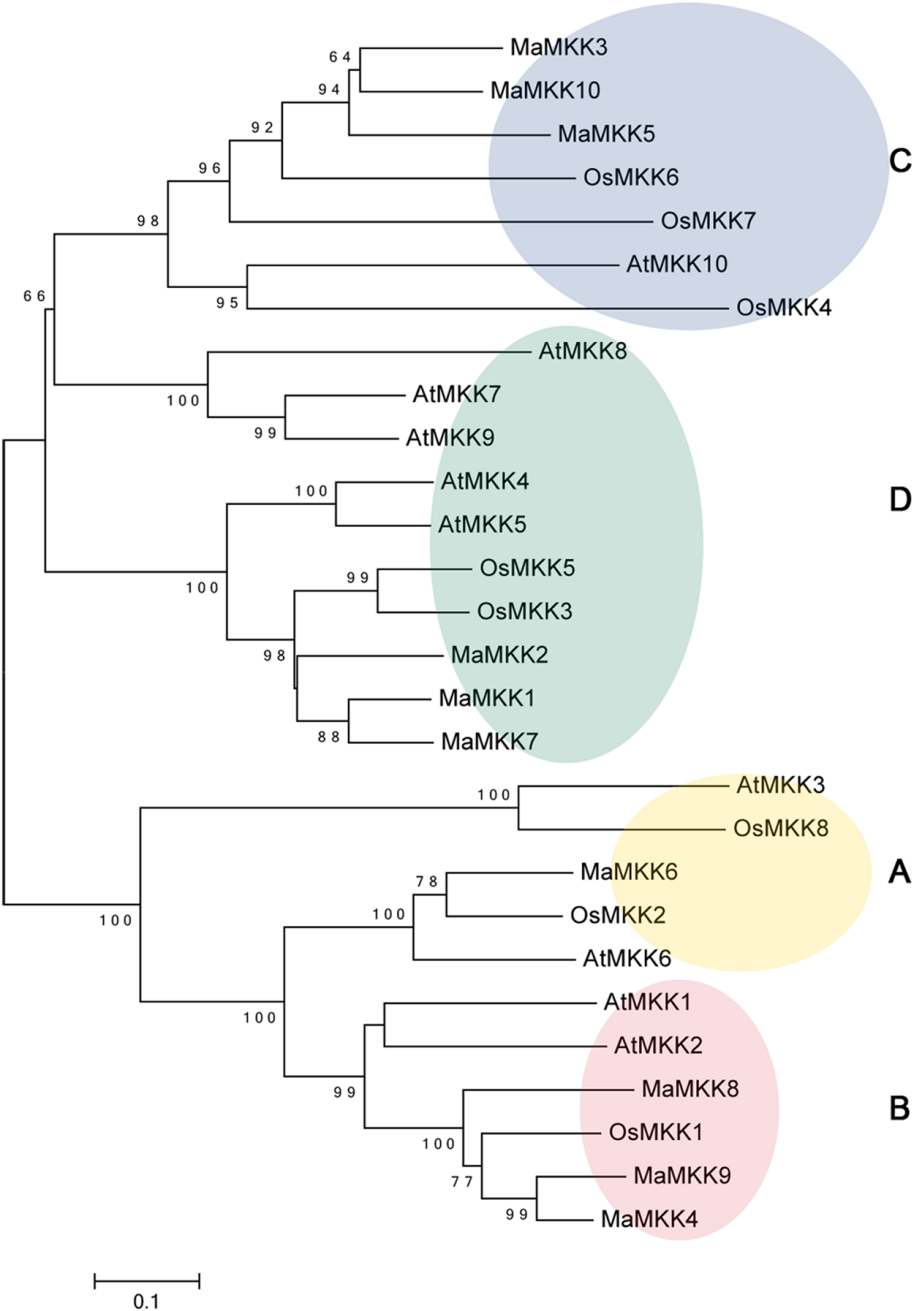
Phylogenetic analysis of MAPKKs from banana, rice and Arabidopsis. The Neighbor-joining (NJ) tree was constructed using ClustalX 2.0 and MEGA 5.0 software with 1000 bootstrap replicates.

**Figure 2:**
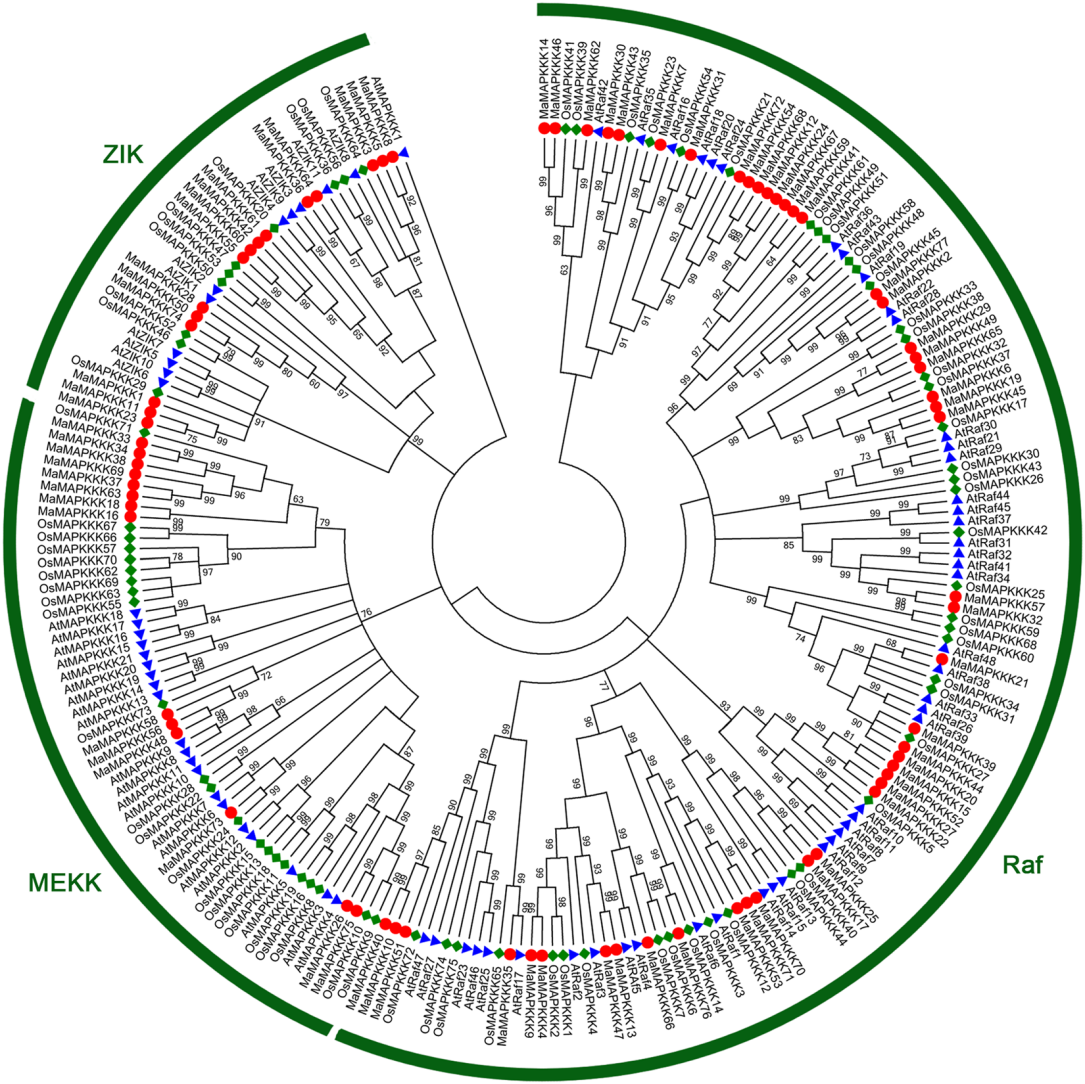
Phylogenetic analysis of MAPKKKs from banana, rice and Arabidopsis. The Neighbor-joining (NJ) tree was constructed using ClustalX 2.0 and MEGA 5.0 software with 1000 bootstrap replicates. Signs of different shapes represent MAPKKK proteins from banana (red round, Ma), rice (green rhombus, Os) and Arabidopsis (blue triangle, At).

To investigate the evolutionary relationship of the MAPKK and MAPKKK families, neighbor-joining (NJ) trees were constructed with MAPKK and MAPKKK proteins from banana, Arabidopsis and rice (Figs 1 and 2). Based on the results of phylogenetic tree, the MAPKK proteins were divided into four groups of A, B, C and D. The MAPKKK proteins were grouped into 3 clusters, named as MEKK, ZIK and Raf. Raf subfamily is large with 48 MaMAPKKK members, whereas the MEKK and ZIK subfamilies each contain less than 15 MaMAPKKK proteins, suggesting that the diversified MAPKKK family in banana may had different functions. As expected, the MAPKK and MAPKKK proteins from banana generally had closer relationships with the proteins from rice than that from Arabidopsis, which is consistent with the current understanding of plant evolutionary history. Some orthologs of MAPKK and MAPKKK between banana, rice and Arabidopsis have also identified from the phylogenetic analysis, which implied that some ancestral *MAPKKK* and *MAPKK* genes had existed prior to the divergence of banana and Arabidopsis.

### Conserved motifs and gene structure analyses of banana MAPKK and MAPKKK families

**Figure 3:**
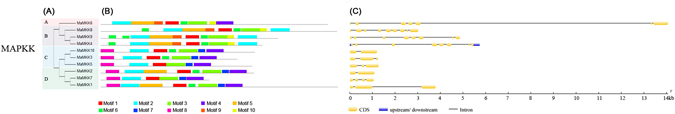
The phylogenetic relationship (**A**), conserved motifs (**B**) and gene structure analyses (**C**) of banana MAPKKs. All motifs were identified by MEME database. The gene structures were drawn using GSDS database. The blue boxes, yellow boxes, and the black lines indicate upstream/downstream, exons and introns, respectively.

**Figure 4:**
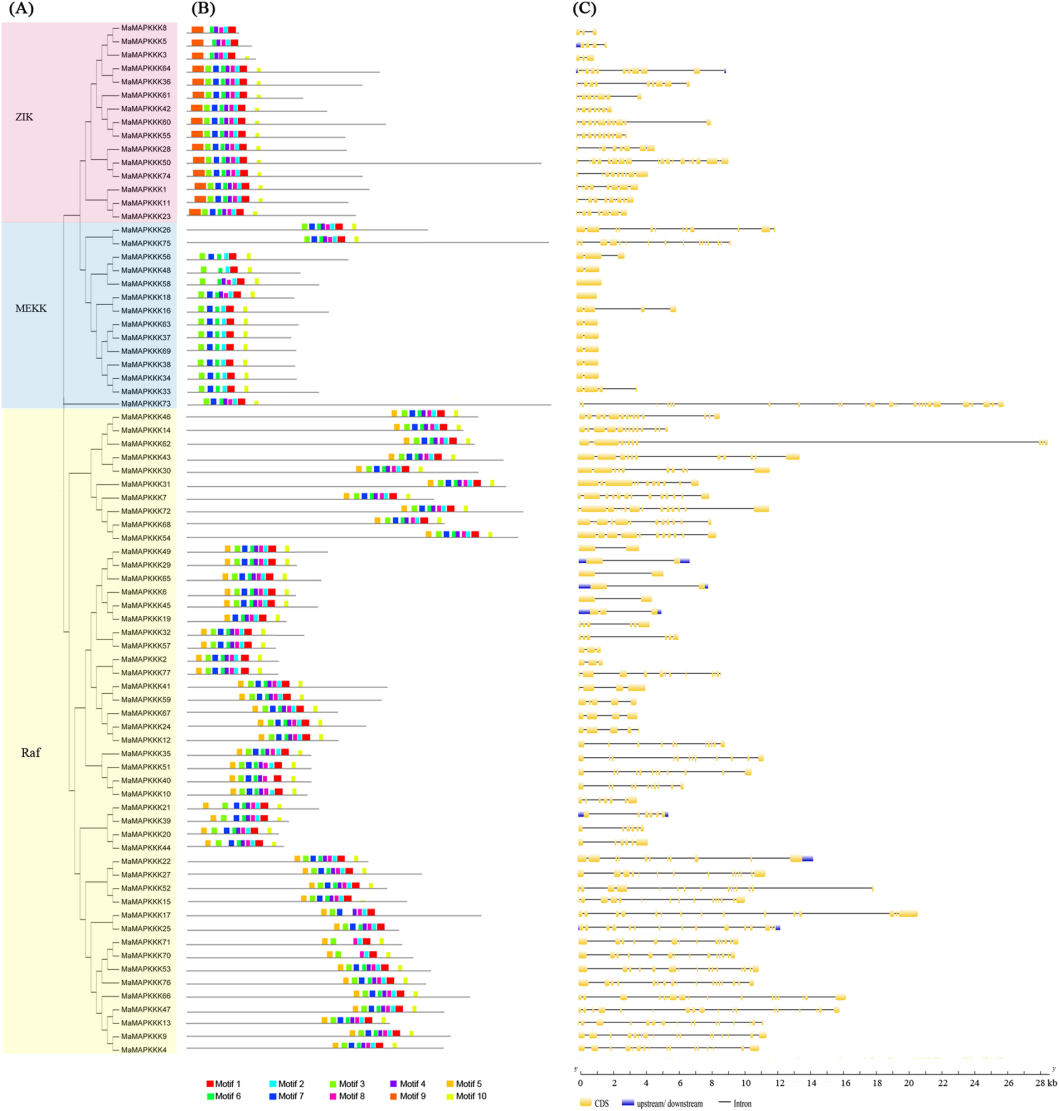
The phylogenetic relationship (**A**), conserved motifs (**B**) and gene structure analyses (**C**) of banana MAPKKK family. All motifs were identified by MEME database. The gene structures were drawn using GSDS database. The blue boxes, yellow boxes, and the black lines indicate upstream/downstream, exons and introns, respectively.

In order to comprehend the evolution of the MAPKK and MAPKKK proteins, 10 conserved motifs in each of the two family proteins were identified with MEME and InterPro database (Figs 3 and 4, Supplementary Table S2). For the MAPKK family, the protein kinase domain of Motifs 1–3 was obtained by all the 10 MAPKKs. The Motifs 7 and 8 were obtained by groups C and D, whereas the Motifs 9 and 10 were obtained by groups A and B. For the MAPKKK family, almost all the proteins harbored the protein kinase domain of Motif 1–3. The Motif 9 and Motif 5 were obtained by ZIK and Raf subfamily, respectively. The results of motif analyses showed that most conserved motifs existed in the same subgroup, indicating that the classification of MAPKKK and MAPKK families was supported by motif analyses.

Exon-intron organizations of the *MAPKK* and *MAPKKK* families were also investigated to get insight into their gene structural evolution (Figs 3C and 4C). For the *MAPKK* family, the group A and B have more exons with more than 7, whereas group C and D are intronless with less than 4 exons (Fig. 3C). For the *MAPKKK* family, most of *MEKK* subfamily genes contain less than 4 exons, while the genes with more than 9 exons were most grouped in the *ZIK* and *Raf* groups (Fig. 4C). These results implied that *MAPKKK* and *MAPKK* members in the same group have similar gene structures, which might be correlated with the gene evolution.

### Expression profiles of *MAPKK* and *MAPKKK* genes in different organs of two banana varieties

**Figure 5:**
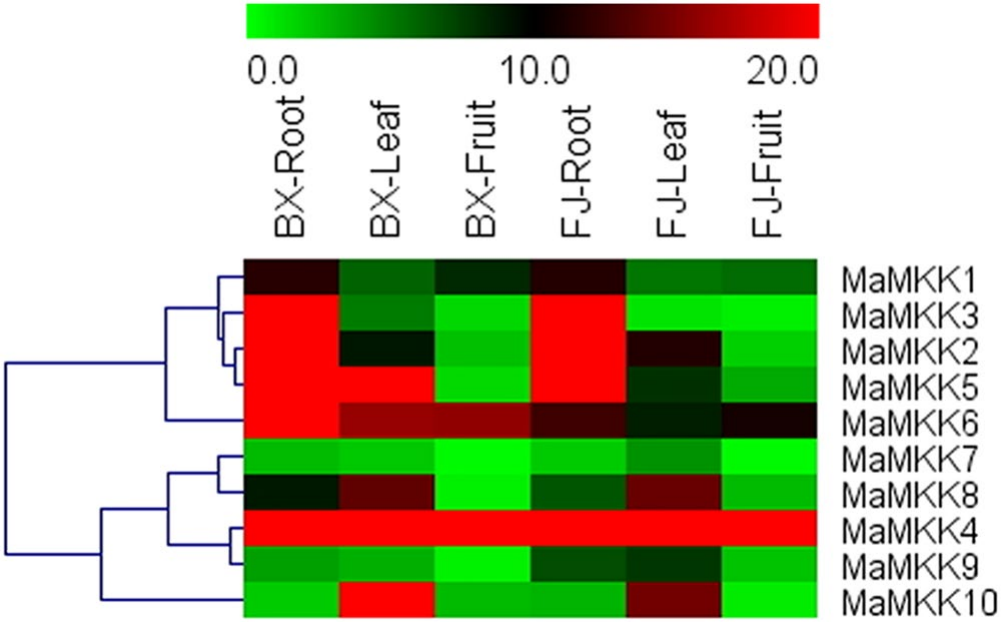
Expression profiles of banana *MAPKKs* in roots, leaves and fruits of BX and FJ. The heat map was constructed according to the FPKM value of banana RNA-seq data. Changes in gene expression are shown in color as the scale.

**Figure 6:**
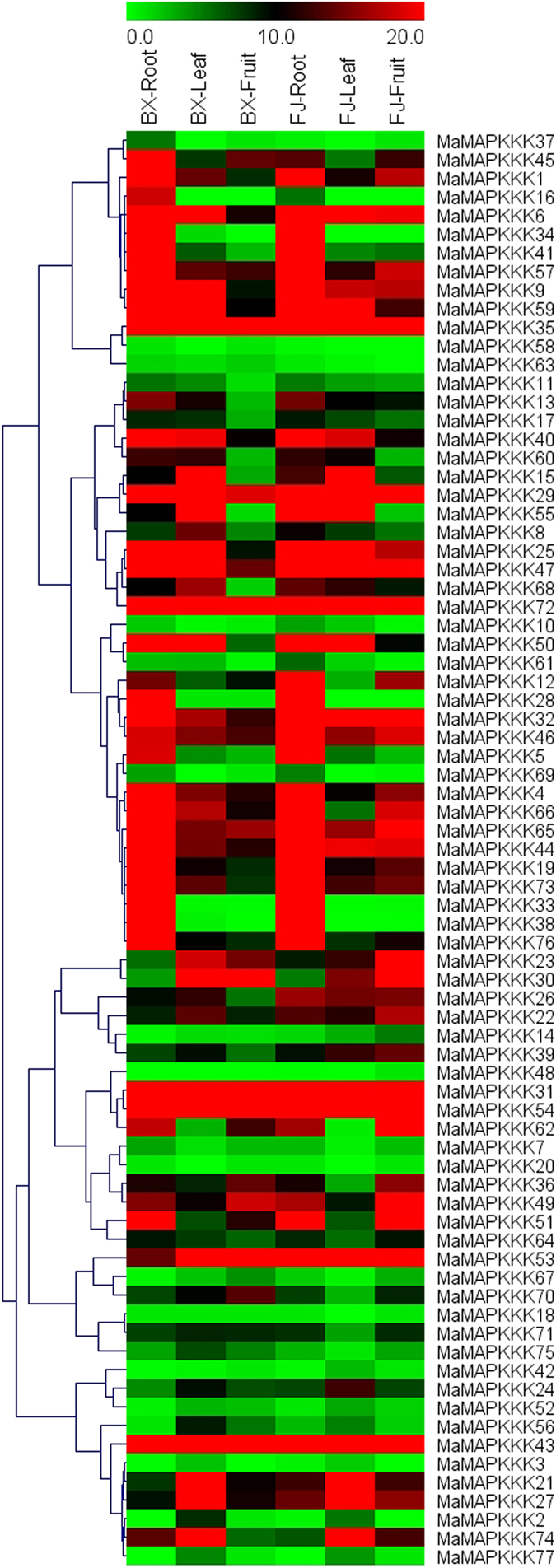
Expression profiles of banana *MAPKKKs* in roots, leaves and fruits of BX and FJ. The heat map was constructed according to the FPKM value of banana RNA-seq data. Changes in gene expression are shown in color as the scale.

In order to examine the organ expression profiles of *MAPKK* and *MAPKKK* genes in banana, samples from roots, leaves and fruits of BX and FJ were collected for RNA-seq analysis (Figs 5 and 6, Supplementary Table S3). For *MAPKKK* family, there were less genes with high expression levels (value > 10) in roots and fruits in BX (53.25% and 36.36%, respectively) than that in FJ (61.04% and 49.35%, respectively).

Comparing the organ expression profiles between BX and FJ, most of *MAPKK* and *MAPKKK* genes showed similar organ expression patterns in the two banana varieties, with more genes showed high expression in roots than in leaves and fruits. Additionally, 18 genes (*MaMKK4*, *MaMAPKKK-4*, -*6*, -*29*, -*31*, -*32*, -*35*, -*40*, -*43*, -*44, -46, -47, -53, -54, -57, -59, -65, -72*) showed high transcripts (value > 10) in all the tested organs of BX and FJ. These results indicated that these genes may play important roles in the organ development of banana. In contrast, 14 genes (*MaMKK7, MaMAPKKK-3, -7, -10, -18, -20, 42, -48, -52, -58, -63, -67, -69, -77*) displayed low transcript abundance (value < 5) in both BX and FJ. Besides, some *MAPKK* and *MAPKKK* genes had differential expression patterns between the two banana varieties, such as *MaMAPKKK-1*, *-6*, *-9, -19*, *-22*, *-25*, *-26*, *-39*, *-66*, *-73* showed low expression levels in BX fruits, while had high expression levels in FJ fruits, suggesting their differential roles in different organs of the two banana varieties. Taken together, the organ expression patterns of *MAPKK* and *MAPKKK* genes in two different varieties may provide valuable information for further investigation of organ development and function.

### Expression profiles of *MAPKK* and *MAPKKK* genes in different stages of fruit development and ripening of two banana varieties

**Figure 7:**
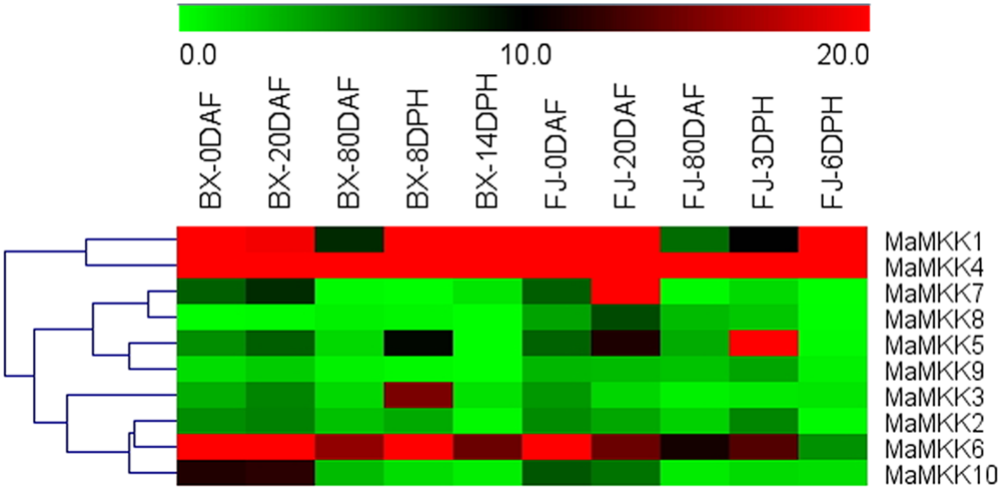
Expression profiles of banana *MAPKKs* in different stages of fruit development and ripening in BX and FJ varieties. The heat map was constructed according to the FPKM value of banana RNA-seq data. Changes in gene expression are shown in color as the scale.

**Figure 8:**
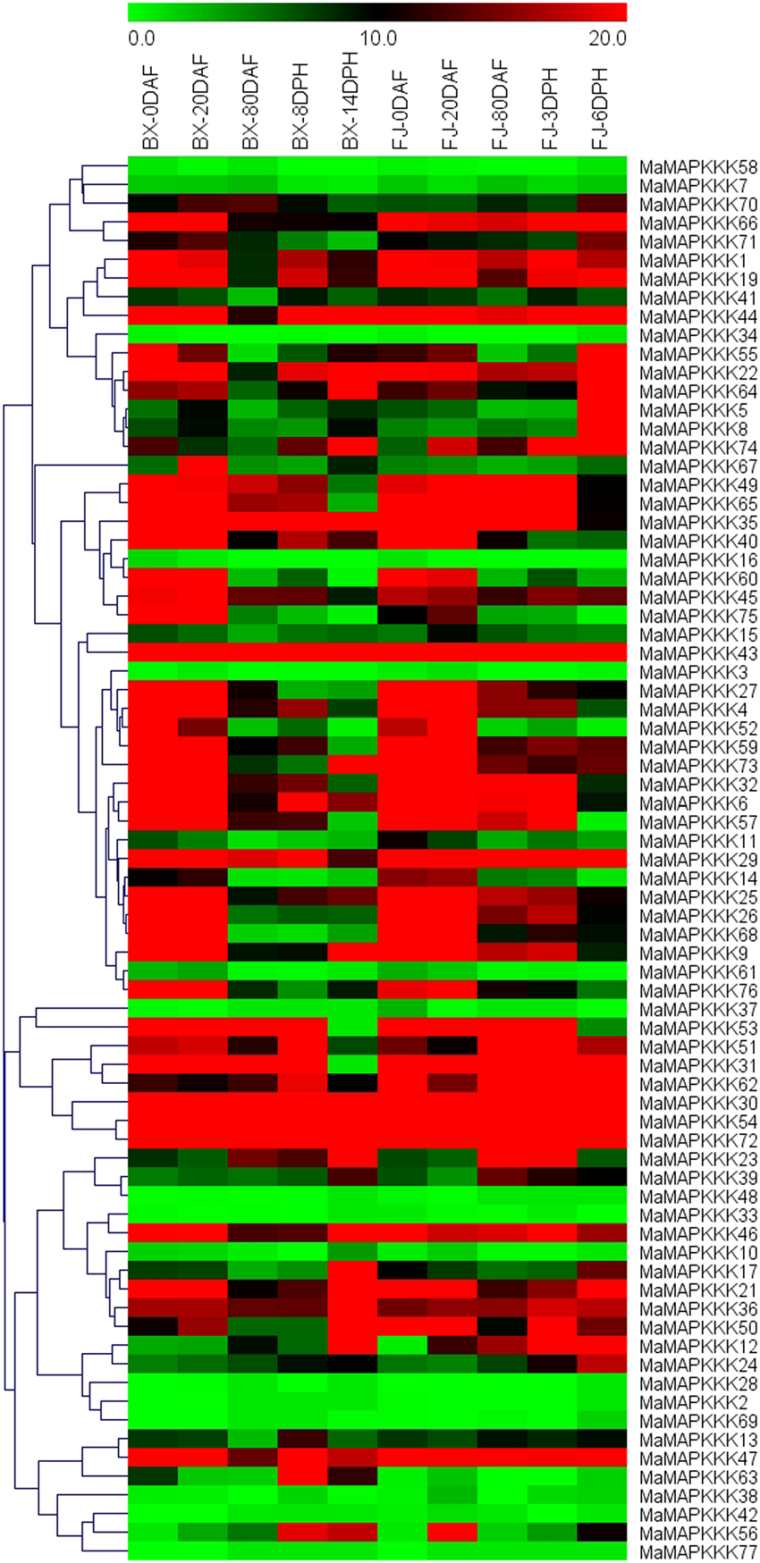
Expression profiles of banana *MAPKKKs* in different stages of fruit development and ripening in BX and FJ varieties. The heat map was constructed according to the FPKM value of banana RNA-seq data. Changes in gene expression are shown in color as the scale.

To get some clues on the roles of *MAPKKK-MAPKK* genes in fruit development and ripening of banana, expression patterns of these genes were detected in fruits sampled from 0, 20, and 80 DAF of the BX and FJ varieties, 8 and 14 DPH of fruits in BX, and 3 and 6 DPH of fruits in FJ (Figs 7 and 8; Supplementary Table S4).

Form the transcriptomic data, the *MAPKK* and *MAPKKK* families genes showed similar expression patterns in the two varieties, with more highly expressed genes (value > 10) at 0 and 20 DAF than that of the other stages. There are more *MaMAPKKKs* with high expression levels (value > 10) at 0 DAF (58.7% in BX and 56.0% in FJ) and 20DAF (58.7% in BX and 60.0% in FJ) than that of subsequent stages (80DAF, 37.3%; 8DPH, 45.7%; 14DPH, 41.3% in BX, 80DAF, 50.6%; 3DPH, 52.0%; 6DPH, 48.0% in FJ) in BX and FJ. Also, similar expression patterns for *MAPKK* genes were also observed in BX and FJ. The results indicated that these genes played a vital role in the early fruit developmental stages of 0 and 20 DAF in BX and FJ.

Additionally, there were more genes that showed high expression levels in FJ than that in BX at 80 DAF, 3/8 DPH and 6/14 DPH for *MAPKKK* family. For example, the ratio of *MaMAPKKK* genes in FJ with high expression levels (value > 10) was more at 80 DAF (50.6%), 3 DPH (52.0%) and 6 DPH (48.0%) than that of the same stages of BX (37.3%, 45.7% and 41.3%, respectively). These findings implied that these genes had more significant transcriptional responses in FJ than in BX during the post-harvest ripening.

A total of 15 *MAPKKK* and *MAPKK* genes, including *MaMKK4*, and *MaMAPKKK-6, -21, -29, -30, -35, -36, -43, -44, -46*, *-47*, *-54, -62, -66, -72*, showed high expression levels (value > 10) during all the tested stages in both BX and FJ, indicating their extensive and vital roles during fruit developmental and ripening processes.

### Expression profiles of *MAPKK* and *MAPKKK* genes under cold, salt, and osmotic stresses in two banana varieties

**Figure 9:**
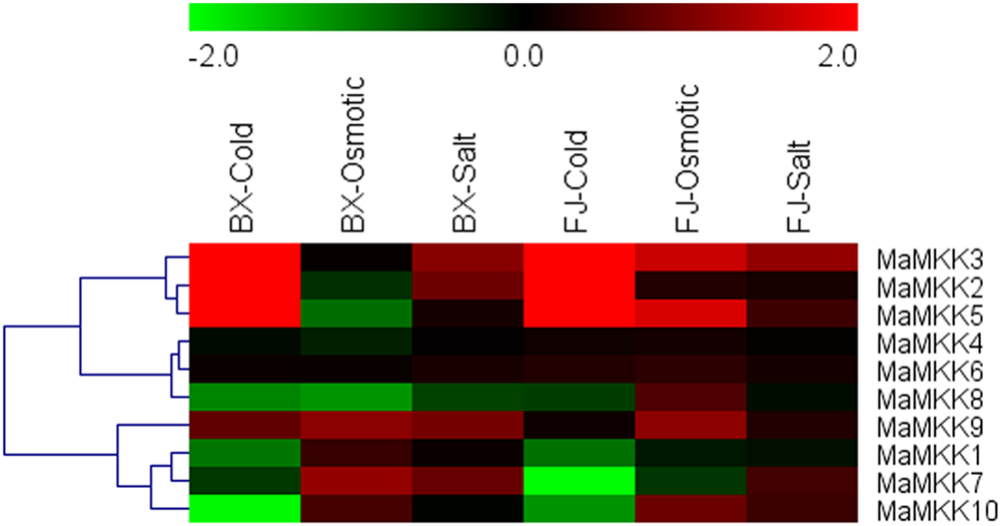
Expression profiles of banana *MAPKKs* in response to cold, osmotic and salt treatments in BX and FJ varieties. Log2 based FPKM value was used to create the heat map. Changes in gene expression are shown in color as the scale.

**Figure 10:**
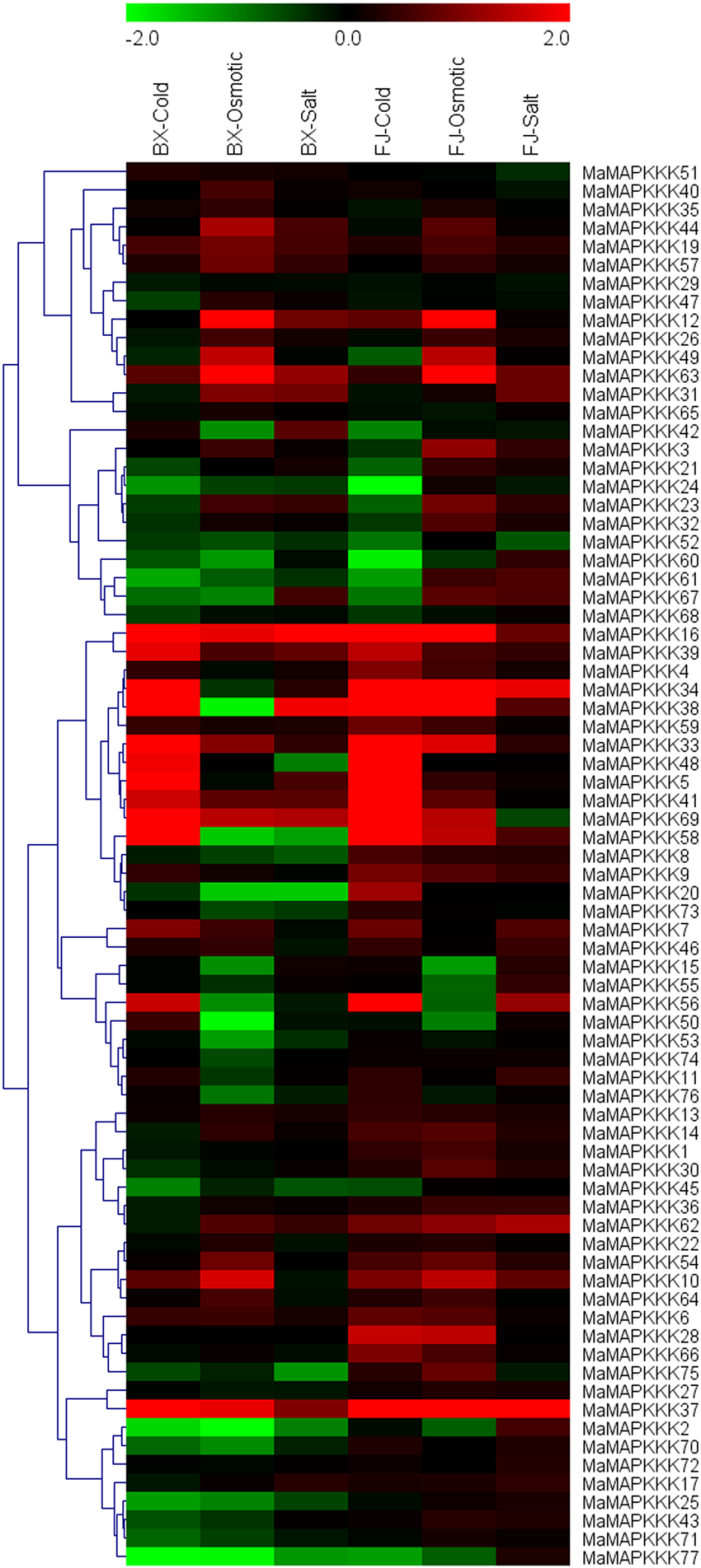
Expression profiles of banana *MAPKKKs* in response to cold, osmotic and salt treatments in BX and FJ varieties. Log2 based FPKM value was used to create the heat map. Changes in gene expression are shown in color as the scale.

To seek insights into the roles of *MAPKKK-MAPKK* genes in banana response to abiotic stress, banana seedlings of BX and FJ under cold, osmotic and salt treatments were sampled to detect gene expression patterns (Figs 9 and 10; Supplementary Table S5).

For *MAPKK* family, 50.0%, 60.0% and 80.0% genes were upregulated by cold, osmotic and salt stress in BX, whereas 60.0%, 80.0% and 85.0% genes were upregulated in FJ. Among them, 30.0%, 20.0% and 10.0% genes were significantly upregulated (Log2 based FPKM >1 and *P*-value < 0.05) by cold, osmotic and salt stress in BX, whereas 30.0%, 30.0% and 10.0% genes were significantly upregulated (Log2 based FPKM >1 and *P*-value < 0.05) in FJ. For *MAPKKK* family, there were 43.4%, 48.7% and 59.2% genes upregulated under cold, osmotic and salt stress in BX, while 65.8%, 76.3% and 82.9% genes upregulated under relative abiotic stress in FJ. Among them, 17.1%, 13.2% and 2.6% genes were significantly induced (Log2 based FPKM >1 and *P*-value < 0.05) by cold, osmotic and salt stress in BX, whereas in FJ the ratio of significantly induced (Log2 based FPKM >1 and *P*-value < 0.05) genes were 19.7%, 15.8% and 5.3% under each stress. These results indicated that the ratio of *MAPKK* and *MAPKKK* genes upregulated by abiotic stress were higher in FJ than in BX, implying that the MAPK cascade may be more active in FJ than in BX in response to abiotic stress.

Five (MaMAPKKK-20, -28, -62, -66, -75), 11 (MaMKK-2, -3, -5, -8, and MaMAPKKK-28, -34, -38, -58, -61, -67, -75), and 8 (MaMPKKK-2, -7, -10, -56, -58, -60, -61, -77) genes were upregulated by cold, osmotic and salt treatment respectively in FJ, whereas they showed downregulation or no changes in BX. Besides, 3 genes (MaMAPKKK-1, -8, -30) showed upregulation under all abiotic stress treatments in FJ, whereas they showed downregulation or no changes in BX. In addition, 3 genes (MaMAPKKK-4, -20, -28), 6 genes (MaMKK-3, -5, MaMAPKKK-28, -34, -38, -58) and 2 genes (MaMAPKKK-34, -56) were upregulated significantly (Log2 based FPKM >1 and P-value < 0.05) in FJ after cold, osmotic and salt treatment respectively, whereas downregulated or no changes in BX. These results indicate that these genes may uniquely function on the tolerances of FJ to abiotic stress.

### Validation of the differentially expressed *MAPKK* and *MAPKKK* genes by qRT-PCR analysis

**Figure 11:**
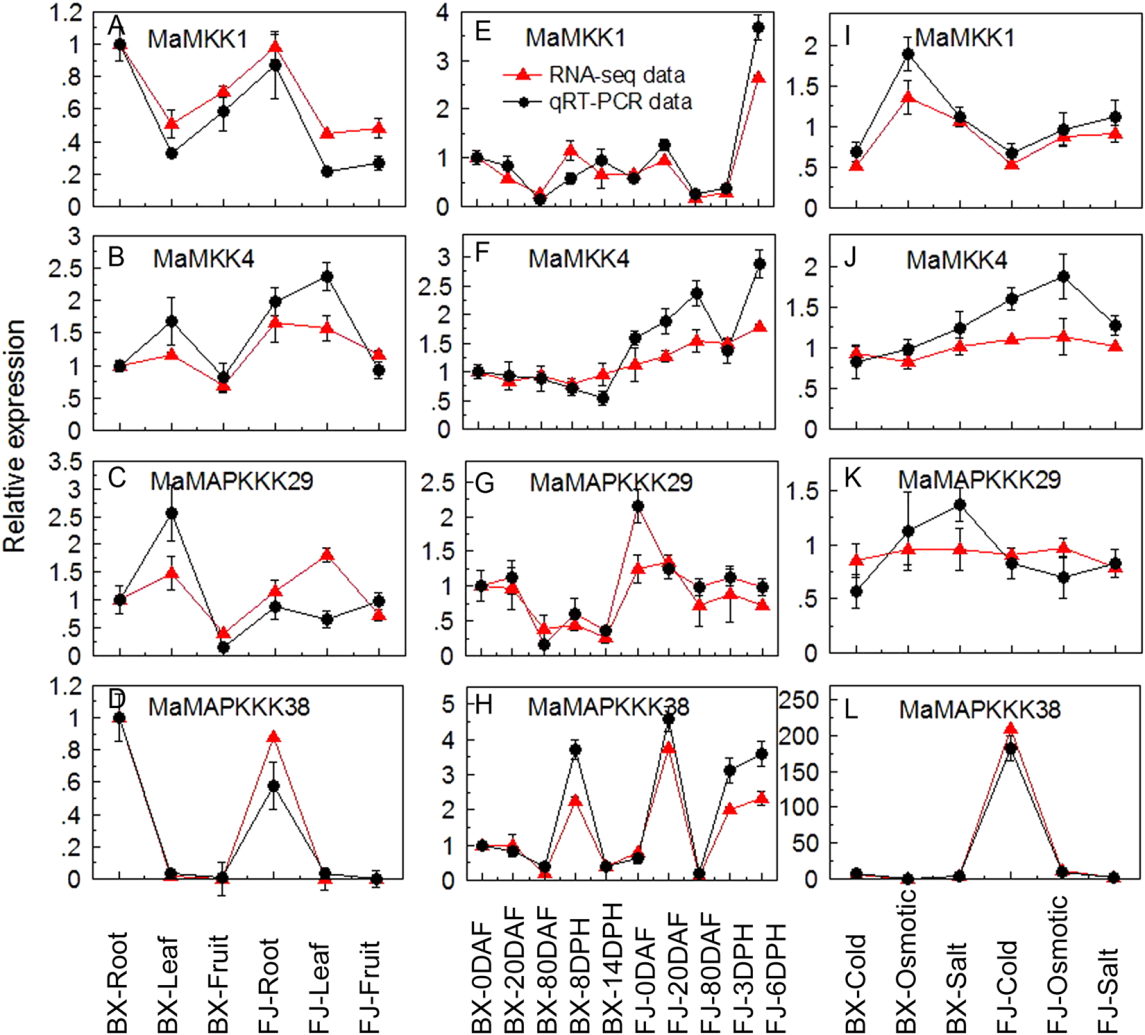
qRT-PCR analysis of relative expression of *MaMKK* and *MaMAPKKK* genes in BX and FJ. (**A**–**D**) Expression patterns of *MaMKK1*, *MaMKK4*, *MaMAPKKK29* and *MaMAPKKK38* in different organs. The mRNA fold difference was relative to that of BX-root samples used as calibrator. (**E**–**H**) Expression patterns of *MaMKK1*, *MaMKK4*, *MaMAPKKK29* and *MaMAPKKK38* in different stages of fruit development and ripening. The mRNA fold difference was relative to that of BX-0DAF samples used as calibrator. (**I**–**L**) Expression patterns of *MaMKK1*, *MaMKK4*, *MaMAPKKK29* and *MaMAPKKK38* in response to cold, osmotic and salt stresses. The mRNA fold difference was relative to that of untreated samples used as calibrator. Log2 based values were used to display differential expression results. Data are means ± SD of 3 biological replicates.

According to the RNA-seq data, four *MaMKK* and *MaMAPKKK* genes (*MaMKK-1*, *-4* and *MaMAPKKK-29*, *-38*) that showed different expression patterns in different organs, in different fruit development and ripening stages, as well as under abiotic stress treatments of BX and FJ were selected for qRT-PCR analysis to validate the RNA-seq data. After normalization, we found that the majority of selected genes, except for *MaMAPKKK29* in FJ-leaf, *MaMKK1* in BX-14DPH, *MaMKK4* in BX-14DPH, *MaMKK4* after osmotic treatment in BX, and *MaMAPKKK29* after osmotic and salt treatments in BX, showed the same trend and consistent results between RNA-seq data and qRT-PCR data (Fig. 11). These results indicate that RNA-seq data are suitable for supplying the expression patterns of *MAPKK* and *MAPKKK* genes in two banana varieties.

### MAPKK member-mediated network and their co-expression after abiotic stress treatment

**Figure 12:**
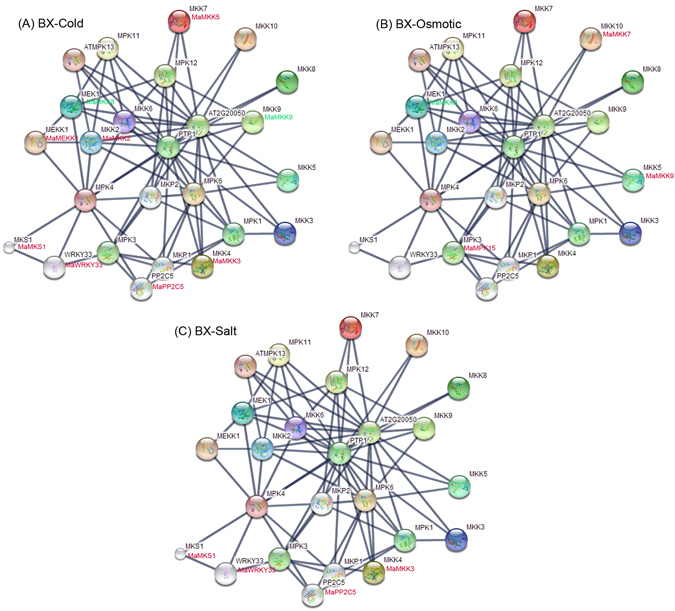
Interaction network and co-expression analyses of banana MAPKKK-MAPKK-MAPK cascades after cold, osmotic and salt treatments in BX and related genes in Arabidopsis. The homologous genes of banana are in parentheses. The genes marked with red show significant upregulation (Log2 based FPKM value >1 and *P*-value < 0.05). The genes marked with green show significant downregulation (Log2 based FPKM value < −1 and *P*-value < 0.05).

**Figure 13:**
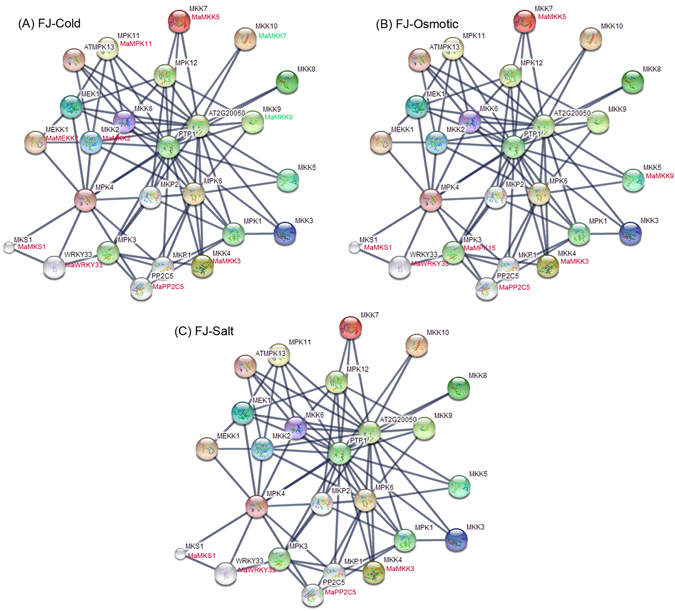
Interaction network and co-expression analyses of banana MAPKKK-MAPKK-MAPK cascades after cold, osmotic and salt treatments in FJ and related genes in Arabidopsis. The homologous genes of banana are in parentheses. The genes marked with red show significant upregulation (Log2 based FPKM value >1 and *P*-value < 0.05). The genes marked with green show significant downregulation (Log2 based FPKM value < −1 and *P*-value < 0.05).

In order to better under the biological function of MAPKKK-MAPKK-MAPK signaling cascades, the interaction networks and co-expression of 10 MAPKKs were investigated based on experimentally validated interactions and transcriptomic data (Figs 12 and 13; Supplementary Table S6). An Arabidopsis MAPKK-mediated interaction network was created and 25 interactive proteins (with high confidence; score >0.9) were identified with STRING database. Then, homologs of these 25 proteins in banana were identified with reciprocal BLASTP algorithm and the expression patterns of these genes in BX and FJ under abiotic stress were extracted from RNA-seq data sets.

Under cold stress in BX, 2 gene pairs *MKK2:MaMKK2-MEKK1:MaMEKK1* and *MKS1:MaMKS1-WRKY33:MaWRKY33* showed co-expression of uniform upregulation (Fig. 12A). Under cold stress in FJ, 3 gene pairs *MKK2:MaMKK2-MEKK1:MaMEKK1*; *MKS1:MaMKS1-WRKY33:MaWRKY33* and *MKK2:MaMKK2-MPK11:MaMPK11* showed uniform upregulation (Log2 based FPKM >1 and *P*-value < 0.05) (Fig. 13A). Under Osmotic treatment, no gene pair was found to be co-expression in BX, whereas 4 genes pairs (*MKS1:MaMKS1-WRKY33:MaWRKY33*; *MPK3:MaMPK15-WRKY33:MaWRKY33*; *MPK3:MaMPK15-PP2C5:MaPP2C5* and *MPK3:MaMPK15-MKK4:MaMKK3*) had upregulated co-expression in FJ (Log2 based FPKM >1 and *P*-value < 0.05) (Figs 12B and 13B). Under salt stress, one gene pairs *MKS1:MaMKS1-WRKY33:MaWRKY33* showed upregulated co-expression in both BX and FJ (Log2 based FPKM >1 and *P*-value < 0.05) (Figs 12C and 13C). Additionally, one gene pairs *MEK1:MaMKK8-MEKK1:MaMEKK1* had negative correlation in the gene expression in BX (Fig. 12A). Collectively, the interaction network and co-expression analyses indicated the crucial roles of MAPKK member-mediated network in stress signaling, and more gene pairs were uniformly up-regulated in FJ than in BX in response to the cold and osmotic stresses.

## Discussion

The MAPKKK-MAPKK-MAPK signaling cascade plays a vital role in regulating plants developmental processes and responses to environmental stresses^3–5^. Investigation of the core regulatory network in the pathway would advance our understanding of the function of MAPK signaling genes. Although some MAPK signaling related genes had studied in many plants, less information is known about the MAPKK and MAPKKK families in banana. Herein, a total of 10 MAPKK and 77 MAPKKK proteins was identified from banana genome, which was classified into 4 and 3 subgroups respectively according to the phylogenetic relationship (Figs 1 and 2). This classification is consist with previous studies of MAPKK and MAPKKK families in Arabidopsis, rice, cucumber and *Brachypodium distachyon* ^3, 18–20, 25, 28^. Moreover, the phylogenetic classification of MAPKK and MAPKKK was also supported by conserved motif and gene structure analyses (Figs 3 and 4). Conserved motif analyses showed that all the MAPKKK-MAPKK proteins had protein kinase domains, and each subfamily shared similar motifs. These typical characteristics of these two gene families were also observed in other plants, such as Arabidopsis, cucumber and *Brachypodium distachyon* ^3, 18–20, 25, 28^. Gene structure analysis suggested that *MAPKKK* and *MAPKK* gene families harbored similar exon-intron organizations in the same subgroup. Most of the *MEKK* subfamily genes contained less than 4 exons, while the *ZIK* and *Raf* groups harbored more than 9 exons. Accordingly, the gene structure analysis results are also observed in *Brachypodium distachyon* and cucumber^25, 27^. In previous report on rice, the rate of intron loss is faster than the rate of intron gain after segmental duplication^29^. The divergent gene structures in the different subgroups suggested that gene duplication events occurred in ancient years, and then the offspring genes evolved into diverse gene structures with introns loss to achieve different functions. Collectively, conserved motif and gene structure analyses indicated that genes in the same group had similar conserved motifs and exon-intron organizations, suggesting banana *MAPKKs* and *MAPKKKs* in the same group had a closer relationship during the gene evolution process.

Banana is one of the most popular fruits, so the fruit development and ripening processes are vital for banana fruit quality^30^. MAPK signaling pathway has been reported to play important roles in fruit development and ripening of many plant species, including grape, potato and cotton^31–33^; however, there are little reports about the *MAPKKK* and *MAPKK* genes involved in fruit development and ripening process of banana. In this study, we found that more than 87.0% of *MAPKKK* and *MAPKK* genes expressed at the 5 tested development and ripening stages of BX and FJ, among which more than 20.0% genes displayed high transcripts (value > 10) at each stage of the two banana varieties (Figs 7 and 8; Supplementary Table S4). These results imply that *MAPKK* and *MAPKKK* genes are extensively involved in the fruit development and ripening processes of banana.

In addition, the ratio of MAPKK and MAPKKK genes with highly expressed levels (value > 10) at 0 and 20 DAF is more than that of the other stages in both BX and FJ, implying the regulatory role of these genes in the early fruit development process (Figs 7 and 8; Supplementary Table S4). This phenomenon was also found in grape, potato and cotton. *ScFRK1*, a wild potato *MAPKKK* gene, expressed early fruit development stages of the embryo sac and pollen development^32^. In cotton Raf family, 11 *MAPKKK* genes had more highly expressed in 0 day post-anthesis (DPA) ovules than in 3-DPA ovules, indicating these genes involved in the early fruit development stages^33^.

By comparing the expression profiles at different stages of fruit development and post-harvest ripening between BX and FJ, it is observed that *MAPKKK* genes with high transcripts (value > 10) were more in FJ than in BX at 80 DAF, 3/8 DPH and 6/14 DPH, which implied that these genes may be more active in FJ than in BX during fruit post-harvest ripening stages (Figs 7 and 8; Supplementary Table S4). Notably, it is observed that FJ ripened faster than BX in postharvest ripening process, as it took 8 and 14 DPH to reach more green than yellow and full yellow degrees of ripening for BX, while it only need 3 and 6 DPH for FJ, respectively^30, 34^. MAPK signal transduction modules has been reported to play important roles in regulating plants development and fruit ripening. Three *MAPKs* were significantly up-regulated during litchi pericarp maturation^35^. In cotton, 32 of 78 *MAPKKK* genes showed high expression after anthesis^33^. Notably, it is reported that MAPK cascades are involved in ethylene signaling pathway, which plays a crucial role in plant fruit development^36^. Exposure of banana fruits to ethylene improved the ripening process of softening, aroma production and reach yellow degree^37^. Several *MAPKs* showed high expression during ethylene-induced banana fruit ripening, which suggests that MAPKs are involved in the ethylene signal transduction pathway^26^. Therefore, these findings suggested that MAPK cascades may positively regulate banana fruit development and ripening process.

As an tropical crop with shallow roots, permanent green canopy, and rapid growth rate, banana always subjected to water stress caused by abiotic stress such as cold, drought, or salt^38^. Thus, investigating the mechanism of banana response to abiotic stress is very important for banana breeding. In the present study, we found that many *MAPKKK* and *MAPKK* genes could response to cold, salt, and osmotic treatment in both BX and FJ, indicating that these genes may function in regulating banana resistance to abiotic stress (Figs 9 and 10; Supplementary Table S5). This phenomenon was also found in tomato, cucumber and *Brachypodium distachyon*, which showed the MAPK cascades genes changed significantly under heat, cold, drought and salt treatments^24, 25, 28^.

By comparing the expression patterns of *MAPKKK* and *MAPKK* genes under abiotic stress between BX and FJ, it was clear that more genes were significantly upregulated (Log2 based RPKM value > 1 and *P*-value < 0.05) in FJ than in BX under the three tested treatments (Figs 9 and 10; Supplementary Table S5). Furthermore, from the interaction network and co-expression analyses, more gene pairs were uniformly up-regulated in FJ than in BX in response to the cold and osmotic stresses (Figs 12 and 13; Supplementary Table S6). The B-genome has been considered to be related to tolerance to abiotic stresses. The *M. balbisiana* with the B-genome is demonstrated to have strong resistance to abiotic stress^39, 40^. Moreover, the “ABB” banana genotypes are more tolerant to drought and other abiotic stresses than other genotypes^39, 40^. Thus, the banana varieties based on “ABB” genotype can be used as a crucial genetic resource for crop improvement for abiotic stress. FJ (ABB genotype), containing the B-genome, has been reported to have strong tolerances to abiotic stress^30^. Together, these findings suggest that the high ratio of MAPK cascade genes and gene pairs upregulated by abiotic stress in FJ could contribute to the tolerance of banana to abiotic stress.

In conclusion, genome-wide analyses had identified 10 *MAPKK* and 77 *MAPKKK* genes from banana, and their classification and evolutionary relationships were investigated by phylogenetic analysis, conserved protein motif and gene structure analyses. The expression profile analyses of *MAPKK* and *MAPKKK* genes reveal their involvement in banana fruit development, ripening and responses to abiotic stress. Additionally, comparison of differential expression profiles of *MAPKKK-MAPKK* genes in two banana varieties of BX and FJ suggested that *MAPK* signaling cascade might positively regulate banana fruit ripening and responses to abiotic stress. Furthermore, interaction networks and co-expression analyses demonstrated the strong transcriptional response of *MAPKKK-MAPKK-MAPK* signaling in FJ responding to abiotic stress, further supporting the crucial role of the genes in banana tolerance to abiotic stress. These data will supply valuable information for functional characterization of *MAPKKK-MAPKK* genes, and lay a solid foundation for further researches on banana breeding.

## Methods

### Identification and phylogenetic analyses

The whole banana (*Musa acuminata*) protein sequences were acquired from the banana genome database^41^. The MAPKK and MAPKKK proteins of rice and Arabidopsis were obtained from the RGAP and TAIR databases, respectively. The HMM profiles built with the MAPKK and MAPKKK proteins from rice and Arabidopsis were used as queries to search predicted homolog proteins in the banana dataset using HMMER software, respectively^42^. BLAST algorithm was also used to identify the predicted banana MAPKK and MAPKKK families with related sequences from rice and Arabidopsis as queries. Then, the potential banana MAPKK and MAPKKK proteins were further examined with PFAM and CDD databases. Then banana, rice and Arabidopsis MAPKK and MAPKKK family proteins were aligned using Clustal X2.0, and then the phylogenetic tree was constructed using MEGA 5.0 software with bootstrap values for 1000 replicates, respectively^43^.

### Protein properties and sequence analyses

ExPASy database was used to predict the molecular weight and isoelectric points of the banana MAPKK and MAPKKK family proteins. MEME software V4.10.2 and InterProScan database were used to analyze the conserved protein motif. The GSDS database was used to analyze gene structure. The STRING was used to detect protein-protein interactions with the confidence score >0.9.

### Plant materials and treatments

Two varieties of BaXi Jiao (*Musa acuminate* L. AAA group cv. Cavendish, BX) and Fen Jiao (*Musa* ABB PisangAwak, FJ) were used for this study. BX is widely cultivated in China because of its virtues of long storage and high production. FJ is widely planted in the Hainan province of China because it has tolerance to abiotic stress. BX and FJ banana seedlings at five-leaf stage were acquired from the banana tissue culture center (Danzhou, Institute of Bananas and Plantains, Chinese Academy of Tropical Agricultural Sciences), and then they were planted under the conditions of 28 °C, 70% relative humidity and 200 μmol m^−2^ s^−1^ light intensity in 16 h light/8 h dark cycle. Roots, leaves from five-leaf stage, and fruits of 80 DAF were collected from BX and FJ separately for gene expression analysis in different organs. Fruits of 0 DAF (budding stage), 20 DAF (cutting flower stage) and 80 DAF (harvest stage), were acquired from BX and FJ to analyze the gene expression pattern of fruit development process. The fruits of 8 and 14 DPH in BX and 3 and 6 DPH in FJ were sampled respectively to analyze gene expression profile of post-harvest ripening process, because FJ ripening faster to reaching full yellow degree than BX^30, 34^. For abiotic stress treatments, The five-leaf stage banana seedlings were subjected to 200 mM mannitol or 300 mM NaCl for 7 days, and 4 °C for 22 h, respectively, to study gene expression in response to osmotic, salt and cold stresses.

### Transcriptomic analysis

Plant RNA extraction kit (TIANGEN, China) was used to extract total RNA, then cDNA libraries were constructed and sequencing were performed with an Illumina GAII following manufacturer’s instructions with 5.34X sequencing depth and two replicates for each sample. The raw data generated from the libraries was deposited in NCBI-SRA database (accession number: PRJNA343716). The transcriptome mapping and assemblies were performed by Tophat v.2.0.10 and Cufflinks^44^. Reads Per Kilobase of exon model per Million mapped reads (FPKM) were considered as gene expression levels. DEGseq was used to identify differentially expressed genes^45^.

### qRT-PCR Analysis

Changes in the expression of *MaMKK* and *MaMAPKKK* genes in different organs, different stages of fruit development and ripening, and response to abiotic stresses of cold, osmotic and salt were detected by quantitative real-time polymerase chain reaction (qRT-PCR) analysis using SYBR® Premix Ex Taq™ (TaKaRa, Shiga, Japan) chemistry on a Stratagene Mx3000 P (Stratagene, CA, USA) instrument. Primers with high specificity and efficiency determined by melting curve analysis and agarose gel electrophoresis were selected to perform quantification assay (Supplementary Table S7), and the amplification efficiencies of primer pairs were between 0.9 and 1.1. *MaRPS2* (HQ853246) and *MaUBQ2* (HQ853254) were used as internal controls to normalize the relative expression of target genes^46^. The relative expression levels of the target genes were assessed based on 2^−ΔΔCt^ method^47^. Each sample contains three replicates.

## Electronic supplementary material

Dataset 1
